# Study on the Effects of the Interphase Region on the Network Properties in Polymer Carbon Nanotube Nanocomposites

**DOI:** 10.3390/polym12010182

**Published:** 2020-01-10

**Authors:** Yasser Zare, Kyong Yop Rhee

**Affiliations:** Department of Mechanical Engineering, College of Engineering, Kyung Hee University, Yongin 446-701, Korea; y.zare@aut.ac.ir

**Keywords:** polymer nanocomposites, percolation threshold, interphase, excluded volume, network fraction

## Abstract

The interphase region around nanoparticles changes the percolation threshold of long and thin nanoparticles, such as carbon nanotubes (CNT) in polymer nanocomposites. In this paper, the effects of the interphase region on the percolation threshold of nanoparticles and the network fraction are studied. New percolation threshold (*φ_P_*) is defined by the role of the interphase in the excluded volume of nanoparticles (V_ex_). Moreover, the influences of filler and interphase size on the percolation volume fraction, the fraction of nanoparticles in the network as well as the volume fraction and relative density of the filler network are investigated. The least ranges of “*φ_P_*” are obtained by thin and long CNT. Similarly, a thick interphase increases the “V_ex_” parameter, which causes a positive role in the percolation occurrence. Also, thin CNT and a thick interphase cause the high fraction of the filler network in the nanocomposites.

## 1. Introduction

**Figure 1:**
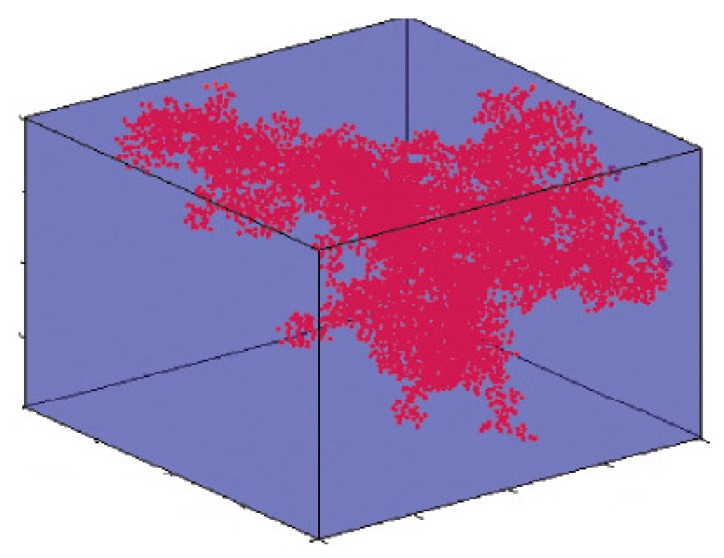
Schematic representation of connected nanoparticles (filler network) in a nanocomposite.

Many researchers have focused on polymer/carbon nanotubes (CNT) nanocomposites because their very low volume fractions show effective mechanical, thermal and chemical properties by preserving low density, transparency and simple processing [1,2,3,4,5,6,7,8,9,10]. The addition of CNT or graphene to polymers forms conductive nanocomposites, which has produced scientific interest in the research communities due to their potential applications in different fields, such as electronics and sensors [11,12,13,14,15,16,17,18,19,20,21,22,23,24,25]. The experiments show the remarkable improvement of electrical conductivity of polymers by the addition of CNT. The electrical conductivity sharply increases when the CNT concentration reaches a certain level as a percolation threshold, which is the minimum volume fraction of filler, above which the nanoparticles form a continuous network structure in the nanocomposite [26,27]. Figure 1 shows a 3D representation for the connected network in a nanocomposite.

**Figure 2:**
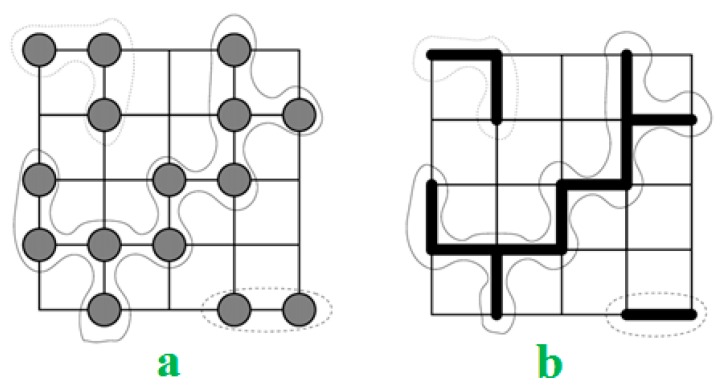
(**a**) Site and (**b**) bond percolation models on a square lattice.

The percolation model generally considers a regular lattice and a random network by randomly “occupying” sites (vertices) or bonds (edges) with a statistically autonomous chance. At a critical threshold, large clusters and long-range connectivity appear. Depending on the technique for finding the random network, the site and bond percolation thresholds are distinguished (Figure 2). In the separate percolation theory, bond percolation is a model on a lattice, which reflects the lattice graph edges as the related objects (Figure 2b).

The interphase region surrounding the nanoparticles as well as the high number of nanoparticles per volume significantly affects the effective properties of nanocomposites [28,29]. The individual nanoparticles have a higher surface area to volume ratio and exhibit a greater number density at the same volume fraction. Accordingly, the total interphase region surrounding the large number of nanoparticles produces an intermediate phase as an interphase, which is different from what occurs with a polymer matrix and nanoparticles. It was well reported that the interphase is formed in the polymer nanocomposites and the properties of the interphase control the mechanical behavior of nanocomposites [30,31,32,33]. However, the interphase is difficultly characterized by experiment, due to its small size and complex nature. Moreover, the conventional models for the mechanical properties of composites cannot account for the interphase role [34,35]. As a result, a number of authors have considered the interphase in the traditional models to improve their estimations for mechanical behavior [36,37,38]. These models can calculate the properties of the interphase by the experimental results of mechanical tests.

The large number of nanoparticles, which generates close stacking along with the interphase region around them, may facilitate the formation of percolated microstructure at lower volume fractions [39]. This pseudo-percolation occurs at a slightly smaller volume fraction of nanoparticles than that of the percolation of nanoparticles without an interphase due to the connection of the interphase region. This assumption agrees with the predicted and experimental volume fractions of the percolation threshold in the nanocomposites with enhanced properties. Celzard et al. [40] suggested that some disagreement between the measured and the predicted values for high aspect ratio and randomly oriented particles may be due to the interactions between the polymer matrix and the particles. This observation confirms the pseudo-percolation of the interphase region in the nanocomposites. However, the interphase percolation in polymer nanocomposites has been briefly studied in the literature.

In our previous paper [41], it was found that the dimensions of the CNT, waviness and interphase layer expressed the percolation threshold in nanocomposites. Moreover, the effective volume fraction of CNT and the fraction of CNT in the network were defined to develop a simple model for the conductivity of nanocomposites. In the current article, the pseudo-percolation in polymer nanocomposites containing long and thin nanoparticles is studied, such as CNT. The effects of the filler and interphase properties on the excluded volume and percolation point are estimated. Also, some equations are applied to show the roles of the filler radius and interphase thickness in the fraction of nanoparticles in the network, the volume fraction of the filler network, and the density of the network. As such, the present paper highlights the effects of filler and interphase properties on the excluded volume of CNT, percolation point, the fraction of nanoparticles in the network, the volume fraction of the filler network and the density of the network, which is different from the previous articles.

## 2. Background

A good method to calculate the CNT volume fraction at percolation in nanocomposites is to use an analytical solution. For this purpose, the volume and excluded volume of nanoparticles are considered. For spherically capped cylindrical rods, the volume is expressed as:

(1) $$V=\pi R^{2}l+(4/3)\pi R^{3}$$

where “*R*” and “*l*” are the radius and length of the nanotubes, respectively. Also, the excluded volume is proposed as the volume around an object into which the center of a similar object cannot enter. For a random distribution of these particles, the excluded volume [42] is defined as:

(2) $$V_{ex}=\frac{32}{3}\pi R^{3}[1+\frac{3}{4}(\frac{l}{R})+\frac{3}{8\pi }<\sin(\theta )>{\left(\frac{l}{R}\right)}^{2}]$$

where “*θ*” is the angle between rods and <sin (*θ*)> is the average value of sin (*θ*). <sin (*θ*)> = π/4 for a random distribution [42], which results in:

(3) $$V_{ex}=\frac{32}{3}\pi R^{3}[1+\frac{3}{4}(\frac{l}{R})+\frac{3}{32}{\left(\frac{l}{R}\right)}^{2}]$$

The analytical solution for the percolation threshold [42] is given by:

(4) $$\phi_{p}=\frac{V}{V_{ex}}$$

Substituting of Equations (1) and (3) into Equation (4), the percolation threshold is defined as:

(5) $$\phi_{p}=\frac{\pi R^{2}l+(4/3)\pi R^{3}}{\frac{32}{3}\pi R^{3}[1+\frac{3}{4}(\frac{l}{R})+\frac{3}{32}{\left(\frac{l}{R}\right)}^{2}]}=\frac{\frac{l}{R}+4/3}{\frac{32}{3}[1+\frac{3}{4}(\frac{l}{R})+\frac{3}{32}{\left(\frac{l}{R}\right)}^{2}]}$$

As mentioned, the interphase region with intermediate properties can contribute to network connectivity. The interphase can increase the excluded volume of each rod by producing a connection before the rods physically touch. To model this, the value of “*R*” changes in the excluded volume to reflect the rod plus interphase, while the volume of rods remains constant. The excluded volume is suggested assuming the interphase as:

(6) $$V_{ex}=\frac{32}{3}\pi {\left(R+t\right)}^{3}[1+\frac{3}{4}(\frac{l}{R+t})+\frac{3}{32}{\left(\frac{l}{R+t}\right)}^{2}]$$

By the new appearance of “*V*_ex_” by interphase thickness, the percolation threshold can be expressed as:

(7) $$\phi_{p}=\frac{V}{V_{ex}}=\frac{\pi R^{2}l+(4/3)\pi R^{3}}{\frac{32}{3}\pi {\left(R+t\right)}^{3}[1+\frac{3}{4}(\frac{l}{R+t})+\frac{3}{32}{\left(\frac{l}{R+t}\right)}^{2}]}$$

In addition to the excluded volume of one individual object, “<*V*_ex_>” as total excluded volume of a system is defined [40] by:

(8) $$<V_{ex}>=N_{c}V_{ex}$$

where “*N*_c_” is the critical number density at percolation point. This term is a dimensional invariant for aligned objects, which can be placed for specific shapes. The total excluded volume is also linked to the percolation volume fraction [40] as:

(9) $$\phi_{p}=1-\exp(\frac{-<V_{ex}>V}{V_{ex}})$$

which suggests a new definition for the percolation threshold as a function of excluded volume. Celzard et al. [40] compared the percolation threshold of particles calculated by the excluded volume model with the experimental measurements from the literature. They calculated the “<*V*_ex_>” for high aspect ratio and randomly oriented rods as 1.4. Therefore, Equation (9) can be given by:

(10) $$\phi_{p}=1-\exp(\frac{-1.4V}{V_{ex}})$$

Assuming the role of the interphase in the percolation threshold of nanoparticles, the “$\phi_{p}$” can be shown as a function of the interphase thickness by substituting of Equations (1) and (6) into Equation (10) as:

(11) $$\phi_{p}=1-\exp\left(\frac{-1.4[\pi R^{2}l+(4/3)\pi R^{3}]}{\frac{32}{3}\pi {\left(R+t\right)}^{3}[1+\frac{3}{4}(\frac{l}{R+t})+\frac{3}{32}{\left(\frac{l}{R+t}\right)}^{2}]}\right)$$

Now, the effects of percolating interphase on the different parameters relating to the network are investigated. The percentage of percolated CNT in the nanocomposite changes from 0 to 1. It can be approximately assessed [43] by:

(12) $$f=\frac{\phi_{f}^{1/3}-\phi_{p}^{1/3}}{1-\phi_{p}^{1/3}}$$

where “$\phi_{f}$” is the whole volume fraction of filler. Also, Chatterjee [44] suggested an equation for “*f*” above the percolation threshold of nanoparticles as:

(13) $$f=1-\exp[-A{\left(\frac{\phi_{f}}{\phi_{p}}-1\right)}^{0.474}]$$

where “*A*” is a constant parameter for filler network.

Assuming the “*f*” parameter, the volume portion of the network in the nanocomposite can be calculated by:

(14) $$\phi_{N}=\frac{f\phi_{f}}{1-(1-f)\phi_{f}}≅f\phi_{f}$$

The “*f*” parameter can be replaced by Equations (12) and (13).

Also, the relative density of the filler network in the nanocomposites [45] is defined as:

(15) $$\hat{\rho }=\frac{\rho_{N}}{\rho_{CNT}}=\frac{\pi Nld^{2}}{4L_{1}L_{2}L_{3}}$$

where “*ρ*_N_” and “*ρ*_CNT_” are the density of the network and CNT, respectively and “*d*” is the diameter of nanotubes. Also, “*N*” is the total number of CNT in the unit cell and *L*_1_*L*_2_*L*_3_ is considered as the unit volume of the network. Assuming the aspect ratio of CNT as α = l / d and *L*_1_ = *L*_2_ = *L*_3_ = 10 μm = 10^4^ nm, “$\hat{\rho }$” is expressed as:

(16) $$\hat{\rho }=\frac{\pi \alpha nd^{3}}{4*10^{12}}=\frac{10^{-12}\pi \alpha nd^{3}}{4}$$

where “*n*” is the number of CNT in a unit volume of the network and “*d*” has nm unit.

A simple equation was also proposed between the percolation threshold and aspect ratio of CNT [44] as:

(17) $$\phi_{p}\approx \frac{1}{\alpha }$$

By replacing of Equation (17) into Equation (16), “$\hat{\rho }$” can be stated as:

(18) $$\hat{\rho }=\frac{10^{-12}\pi nd^{3}}{4\phi_{p}}$$

which correlates the relative density of the network to the percolation threshold. The “$\phi_{p}$” can be replaced by Equations (7) and (11).

The waviness decreases the effective length of CNT in nanocomposites [46]. In other words, the waviness weakens the efficient length of CNT, which contributes to percolation threshold, the fraction of CNT in the network, the volume fraction of the filler network and the density of the network. Since the CNT length positively handles the mentioned terms, the waved CNT produce the high percolation threshold, low fraction of CNT in the network, low volume fraction of the filler network and poor network density. Therefore, CNT waviness negatively affects the properties of CNT network in the nanocomposites, which can be considered by the above equations. To consider the waviness in the developed equations, it is enough to use the effective length of waved CNT as the least distance between the two ends of the waved CNT.

## 3. Results and Discussion

### 3.1. Evaluation of Predictions

We use several samples from the literature to verify the predictions of the percolation threshold by Equations (7) and (11). Unfortunately, the authors did not report the experimental data of the fraction of CNT in the network, nor the volume fraction of the filler network and the density of the network. So, we cannot compare the predictions of the suggested equations to the experimental data. Three samples, including polycarbonate (PC)/acrylonitrile butadiene styrene (ABS)/multi-walled CNT (MWCNT) (*R* = 5 nm, *l* ≈ 1.5 μm and $\phi_{p}$ = 0.002) [47], epoxy/single-walled CNT (SWCNT) (R = 1 nm, l ≈ 2 μm and $\phi_{p}$ = 0.0003) [48] and epoxy/MWCNT (R = 8 nm, l ≈ 30 μm and $\phi_{p}$ = 0.0002) [49] are considered. The percolation threshold of these samples is compared to Equation (7). It is found that this equation properly predicts the percolation threshold by the interphase thickness of 3, 0.7 and 2.5 nm for PC/ABS/MWCNT, epoxy/SWCNT epoxy/MWCNT samples, respectively. These values for interphase thickness are reasonable, confirming the predictions of Equation (7). Additionally, two samples including poly(lactic acid) (PLA)/MWCNT (*R* = 15 nm, *l* ≈ 3 μm and $\phi_{p}$= 0.005) [50] and poly(vinyl chloride) (PVC)/MWCNT (R = 8 nm, l ≈ 16 μm and $\phi_{p}$ = 0.0005) [51] are considered. Equation (11) can predict the percolation threshold of these samples using the CNT dimensions. When the details of samples are substituted into Equation (11), the interphase thickness of 5 and 3 nm ar e calculated for PLA/MWCNT and PVC/MWCNT samples, respectively. These levels for interphase thickness are sensible, thus validating the mentioned equation. Therefore, both Equations (7) and (11) properly predict the percolation threshold in polymer CNT nanocomposites.

### 3.2. Parametric Analysis

In this section, the influences of the radius and length of the nanoparticles, as well as interphase dimension on the excluded volume and percolation threshold, are investigated at the first step. Likewise, the roles of nanoparticle and interphase sizes in the fraction of nanoparticles in the network, the volume fraction and relative density of the filler network are assessed and discussed by the expressed equations.

**Figure 3:**
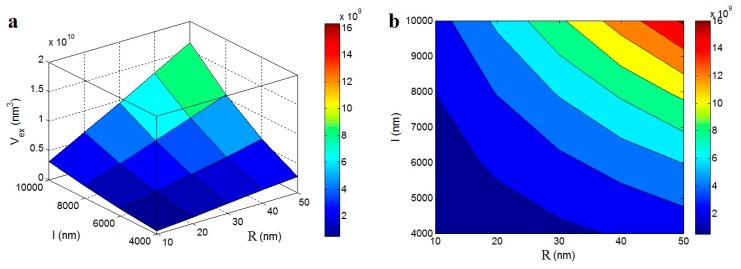
The roles of “*R*” and “*l*” parameters in the excluded volume according to Equation (3): (**a**) 3D and (**b**) contour illustrations.

Figure 3 shows the roles of “*R*” and “*l*” factors in the “*V*_ex_” levels according to Equation (3) by 3D and contour plots. The “*V*_ex_” grows as the “*R*” and “*l*” increase; the highest *V*_ex_ = 16 × 10^9^ nm^3^ is calculated at *R* = 50 nm and *l* = 10,000 nm. However, the “*V*_ex_” decreases to about zero at *R* < 20 nm and l < 5500 nm. Therefore, the “R” and “l” parameters show direct effects on the “*V*_ex_” parameter. The thin and short nanotubes show small, excluded volumes and so, they can be firmly packed and connected. However, the thick and long nanotubes, which produce a big, excluded volume, are constrained in terms of contact. Therefore, the packing of nanoparticles and the interaction among them significantly depends on their dimensions. According to these explanations, the levels of “*R*” and “*l*” parameters affect the percolation threshold of nanoparticles by controlling the excluded volume. The effects of these parameters on the percolation threshold are studied in the following plots.

**Figure 4:**
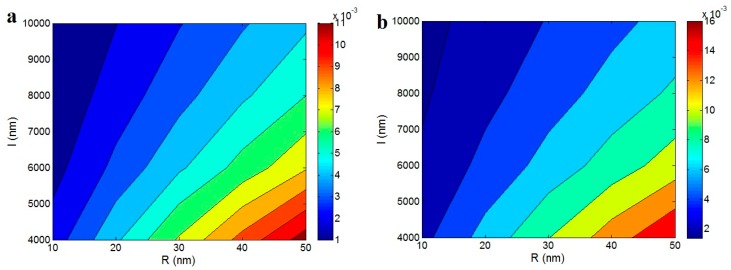
The contour plots for the influences of “*R*” and “*l*” parameters on the percolation threshold of nanoparticles based on (**a**) Equation (5) and (**b**) Equation (10).

Figure 4 illustrates the contour plots for the effects of “*R*” and “*l*” parameters on the percolation threshold of nanoparticles in the nanocomposites based on Equations (5) and (10). The most desirable levels of “$\phi_{p}$” (the lowest ranges) are obtained by the thin and long nanotubes, while thick and small ones observe the highest values of “$\phi_{p}$” (the worst ones). The R = 10 nm and l = 10,000 nm result in the $\phi_{p}$ < 0.002, but R > 45 nm and l < 4500 nm increase the “$\phi_{p}$” to more than 0.01. As a result, the thin and long nanoparticles are perfectly desirable to achieve the lowest level of percolation threshold in the nanocomposites. The packing of nanoparticles considerably affect the percolation threshold. If nanoparticles can be strongly packed (small excluded volume), more of nanoparticles can be connected across a wide area. However, a large excluded volume causes the nanoparticles to be constrained to touch and a long range can be spanned with less objects. As a result, the thin nanoparticles, which decrease the level of excluded volume cause a good packing and a low level for the percolation threshold. Also, the excluded volume more rapidly increases compared to the volume of nanoparticles when the length of the nanotubes enhances. As a result, the large nanotubes obtain a low percolation threshold (Equation (5)). From these remarks, the potential effects of nanoparticles for the polymer reinforcement and electrical conductivity are disclosed.

The nanotubes commonly show a high aspect ratio, which is defined as the length per diameter. Obviously, a high level of aspect ratio (long and thin nanotubes) leads to good conditions for the percolating effect and the formation of the filler network. In addition to a good percolation threshold, the large aspect ratio of the nanoparticles can cause a significant reinforcement in the nanocomposites through a high interfacial area/interaction between the polymer matrix and nanoparticles [52,53].

**Figure 5:**
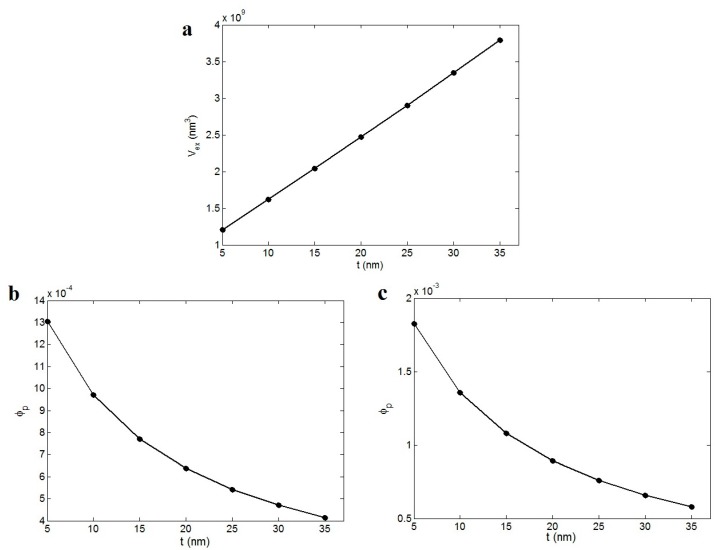
The effects of the interphase thickness on (**a**) excluded volume and the percolation threshold of nanoparticles according to (**b**) Equation (7) and (**c**) Equation (10) at *R* = 10 nm and *l* = 5000 nm.

Figure 5a displays the effect of the interphase thickness on the excluded volume at *R* = 10 nm and *l* = 5000 nm. As observed, the “*V*_ex_” increases from 1.2 × 10^9^ nm^3^ at *t* = 5 nm to 3.8 × 10^9^ nm^3^ at *t* = 35 nm. Accordingly, a direct relation is obtained between the “*V*_ex_” parameter and the thickness of the interphase. Clearly, the variation of “*V*_ex_” by the formation of the interphase around the nanoparticles affects the percolation threshold of nanoparticles in the nanocomposites.

Figure 5b,c show the percolation threshold of nanoparticles in nanocomposites as a function of the interphase thickness according to Equations (7) and (11). The “$\phi_{p}$” decreases by the increment of the interphase thickness. The interphase thickness of 5 nm results in “$\phi_{p}$” levels of 0.0013 and 0.0018 based on Figure 5b,c, respectively. However, *t* = 35 nm causes a decrement in “$\phi_{p}$” to about 0.004 and 0.0006 in Figure 5a,b. Therefore, the interphase plays a positive role in the “$\phi_{p}$” parameter. On the other hand, it is important to note that the properties of the interphase control the level of percolation, i.e., the networking level of nanoparticles in the nanocomposites. As a result, the interphase is an important subject in the nanocomposites. The interphase commonly causes a reinforcement in the polymer nanocomposites, which is indicated in many works in the literature [27,54]. However, the role of the interphase in the percolation of nanoparticles has been less studied in the previous researches. The interphase region around the nanoparticles causes a connection between the nanoparticles before the nanoparticles can physically connect in the polymer matrix. In other words, the interphase accelerates the networking of nanoparticles before actual contact among the nanoparticles by growth of the excluded volume of nanoparticles in nanocomposites. By these explanations, it is concluded that the interphase plays an effective role in the percolation of nanoparticles.

**Figure 6:**
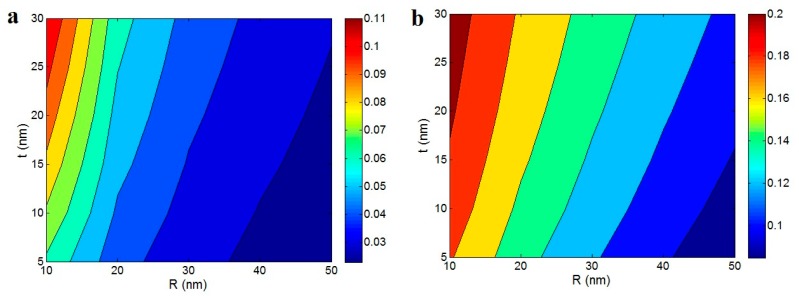
The roles of “*R*” and “*t*” parameters in the fraction of percolating network in the nanocomposites (*f*) according to (**a**) Equation (12) and (**b**) Equation (13) at $\phi_{f}$ = 0.02, *l* = 5000 nm and *A* = 0.02.

Figure 6 also reflects the roles of “*R*” and “*t*” parameters in the fraction of percolating network in the nanocomposites according to Equations (12) and (13) at $\phi_{f}$ = 0.02, *l* = 5000 nm and *A* = 0.02. The smallest level of “*R*” and the largest value of “*t*” obtain the highest fraction of the network. As a result, the best “*f*” is obtained by the thinnest nanoparticles and the thickest interphase. However, it is observed that the thick nanoparticles and thin interphase may lead to the absence of a filler network in the nanocomposites. The best values of “*f*” are obtained as 0.11 and 0.2 by *R* = 10 nm and *t* = 30 nm, while the “*f*” level of zero (absence of the network) is shown in *R* = 50 nm and *t* = 5 nm. Therefore, the sizes of nanoparticles and interphase meaningfully affect the fraction of the networked nanoparticles. It seems that the thick nanoparticles prevent the formation of the filler network in the nanocomposites, due to poor packing. Also, the thin interphase cannot suggest a high level of the filler network in the nanocomposites, because of the low level of excluded volume and high value of percolation threshold.

It was suggested that the number of contacts per particle is directly and inversely related to the excluded volume and particle volume, respectively [55]. The thick nanotubes more increase the volume of nanoparticles than the excluded volume (see Equations (1) and (3)). Therefore, the thick nanoparticles decrease the number of contacts per particle, which prevents network formation. Also, a thin interphase decreases the “*V*_ex_” (Figure 5a), which reduces the contacts among nanoparticles. Accordingly, the observed effects of “R” and “t” parameters on the fraction of percolating network are true. On the other hand, the thin nanoparticles and thick interphase can promote the contacts among the nanoparticles by interfacial interactions, which cause a high level of “*f*”.

**Figure 7:**
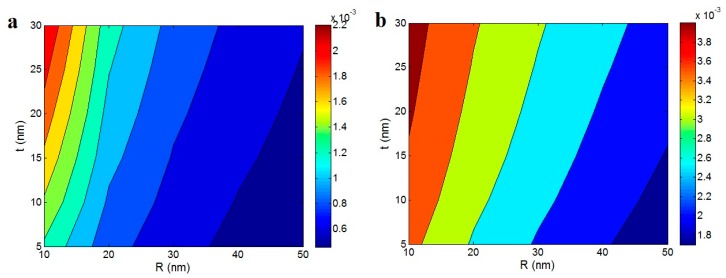
The “$\phi_{N}$” as a function of “*R*” and “*t*” parameters at $\phi_{f}$ = 0.02, *l* = 5000 nm and *A* = 0.02 based on the “*f*” estimations of (**a**) Equation (12) and (**b**) Equation (13).

Figure 7 reveals the influences of “R” and “t” parameters on the “$\phi_{N}$” at $\phi_{f}$ = 0.02, l = 5000 nm and A = 0.02. Expectedly, the “$\phi_{N}$” parameter shows the highest levels by the thinnest nanotubes and the thickest interphase. However, the “$\phi_{N}$” levels predicted using Equation (13) is about two times of Equation (12) at the same conditions for other parameters. For example, the best “$\phi_{N}$” is obtained as about 0.002 at R = 10 and t = 30 in Figure 7a, while $\phi_{N}$ = 0.004 is observed at the same levels of “*R*” and “*t*” parameters in Figure 7b. As a result, Equation (13) predicts the higher values for “$\phi_{N}$” compared to Equation (12). However, both equations predict $\phi_{N}$ = 0 at high “*R*” and low “*t*” values demonstrating the undesirable roles of these ranges in the network fraction. Generally, it is important to incorporate the thin nanoparticles in the nanocomposites by preserving the nanoscale size of nanoparticles during the nanocomposite synthesis. Although the nanoparticles tend to aggregation/agglomeration, the material and processing parameters should be properly chosen to prevent the aggregation/agglomeration and provide the smallest size at the nanoscale in the nanocomposites [56]. Likewise, the interphase can be thickened by the strong interfacial interaction between the polymer matrix and nanoparticles [57,58]. However, the interfacial interaction depends on the compatibility between the polymer matrix and the nanoparticles, which can be improved by significant similarity of surface chemistry of nanofillers and structure of polymer chains. The improvement of compatibility can be obtained by surface treatment of nanoparticles and functionalization of polymer chains or applying a proper compatibilizer [59,60]. The thick interphase can connect the nanoparticles before the nanoparticles may physically connect. Therefore, the connection and networking of nanoparticles can be facilitated by the thick interphase. Also, the thin nanoparticles can promote the interfacial interaction among the nanoparticles and also between the polymer matrix and the nanoparticles, which can provide good conditions for the percolation and networking of nanofiller in the nanocomposites.

**Figure 8:**
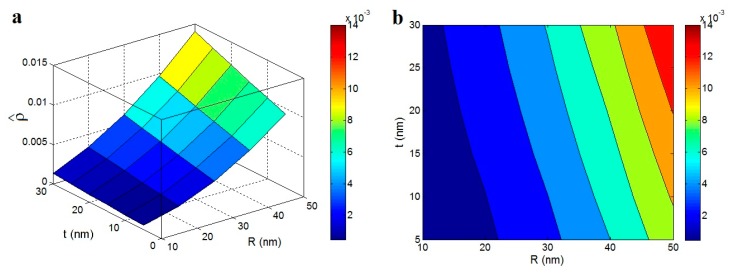
The roles of nanoparticles and interphase dimensions in the relative density of the network by “$\phi_{p}$” predictions of Equation (7) at $\phi_{f}$ = 0.02, *l* = 5000 nm and *n* = 100: (**a**) 3D and (**b**) contour plots.

Finally, the relative density of the network is shown in Figure 8 as a function of the sizes of nanoparticles and interphase by “$\phi_{p}$” predictions of Equation (7) at $\phi_{f}$ = 0.02, *l* = 5000 nm and *n* = 100. It is shown that the greatest density of the network is obtained by the high levels of “R” and “t”, while the low density is calculated by the slight levels of these parameters. As observed, the relative density at *R* = 50 nm and *t* = 30 nm is seventeen times that of its level at *R* < 15 nm and *t* < 15 nm. As a result, both parameters significantly change the level of the network density in polymer nanocomposites. Clearly, the big nanoparticles cause a high density in the nanocomposites, because they occupy a high volume in the network. Also, a thick interphase increases the excluded volume, which produces a dense network in the nanocomposite. Although the interphase thickness shows positive effects on the percolation threshold, network fraction and density, the size of nanoparticles play different roles in the percolation threshold and network properties. Generally, it is concluded that a thick interphase can decrease the percolation threshold and promote the properties of the network in the nanocomposites. Therefore, it is essential to provide a thick interphase between the polymer matrix and the nanoparticles, which finally improves the reinforcing effect of the nanoparticles in the polymer nanocomposites.

## 4. Conclusions

The effect of the interphase region on the percolation threshold of nanoparticles was studied by the role of the interphase in the excluded volume of the nanoparticles. The roles of the filler radius and interphase size in the percolation volume fraction, the fraction of nanoparticles in the network, volume fraction of the filler network and the density of the network were investigated. Thin and short nanotubes produce small excluded volume and so, they can be firmly packed. However, the lowest range of “$\phi_{p}$” is obtained by the thinnest and the longest nanotubes, i.e., by the highest aspect ratio. Also, a direct relation is obtained between the “*V*_ex_” parameter and the thickness of the interphase, which causes a positive effect on the percolation threshold. The interphase region around the nanoparticles produces a connection between nanoparticles before the physical linking of nanoparticles in the polymer matrix, i.e., the interphase accelerates the networking of nanoparticles before an actual contact of nanoparticles. As a result, the properties of the interphase control the level of percolation and networking of nanoparticles in the nanocomposites. The interphase thickness causes the positive effects on the percolation threshold, the fraction and relative density of the network in nanocomposites; but, the size of nanoparticles plays different roles in the percolation threshold and network density. It is necessary to produce a thick interphase through strong interfacial interaction between the polymer chains and CNT, which can decrease the percolation point and promote the properties of the filler network in terms of the fraction and density of the network.
